# Minimizing ED Waiting Times and Improving Patient Flow and Experience of Care

**DOI:** 10.1155/2014/981472

**Published:** 2014-04-14

**Authors:** Assaad Sayah, Loni Rogers, Karthik Devarajan, Lisa Kingsley-Rocker, Luis F. Lobon

**Affiliations:** ^1^Department of Emergency Medicine, Cambridge Health Alliance, 1493 Cambridge Street, Cambridge, MA 02139, USA; ^2^Tufts University School of Medicine, Boston, MA, USA; ^3^Heller School of Social Policy and Management, Brandeis University, Waltham, MA, USA

## Abstract

We conducted a pre- and postintervention analysis to assess the impact of a process improvement project at the Cambridge Hospital ED. Through a comprehensive and collaborative process, we reengineered the emergency patient experience from arrival to departure. The ED operational changes have had a significant positive impact on all measured metrics. Ambulance diversion decreased from a mean of 148 hours per quarter before changes in July 2006 to 0 hours since April 2007. ED total length of stay decreased from a mean of 204 minutes before the changes to 132 minutes. Press Ganey patient satisfaction scores rose from the 12th percentile to the 59th percentile. ED patient volume grew by 11%, from a mean of 7,221 patients per quarter to 8,044 patients per quarter. Compliance with ED specific quality core measures improved from a mean of 71% to 97%. The mean rate of ED patients that left without being seen (LWBS) dropped from 4.1% to 0.9%. Improving ED operational efficiency allowed us to accommodate increasing volume while improving the quality of care and satisfaction of the ED patients with minimal additional resources, space, or staffing.

## 1. Introduction

At the intersection of diagnosis, treatment, and immense patient volumes, the Emergency Department (ED) is arguably the most operationally complex clinical setting of the modern hospital. Unfortunately, it may also be the least understood. From 1995 to 2009, annual ED visits in the US increased by 41% (from 96.5 million to 136.1 million). At the same time, however, the number of hospital Emergency Departments decreased by 27% (from 2,446 to 1,779) [1–3]. Long ED waiting and turnaround times have been shown to decrease both quality outcomes and patient satisfaction [4–6]. Among US Emergency Departments in 2010, only 31% achieved the appropriate triage targets for their patients, while only 48% admitted their patients within 6 hours [7]. 

Like many other EDs around the country, The Cambridge Hospital (TCH) ED suffered from similar patient flow issues including long waits, inefficient processes, and poor patient satisfaction. This paper describes the changes implemented at TCH and the resulting impact on quality, patient flow, and experience of care.

## 2. Methods

We conducted a pre- and postintervention analysis to assess the impact of a patient flow improvement project at TCH, an academic public institution located in Cambridge Massachusetts with an annual ED census of approximately 30,000 patients. Every patient that entered the ED from January 2005 to December 2011 was included as a study participant. The protocol received an Exempt Status from the Cambridge Health Alliance IRB.

In mid-2006, we committed to a system-wide process improvement project aimed at optimizing the ED patient experience by expediting throughput and flow. The Patient Flow Project used modern quality improvement operational methodologies and sought to achieve the following goals:

- to improve patient flow in an effective and patient centered manner;
- to implement evidence-based best practices;
- to utilize improvement methodologies, tools, and measures;
- to utilize a multidisciplinary, multicampus single solution approach;
- to explore the reasons for ED leakage.

**Figure 1:**
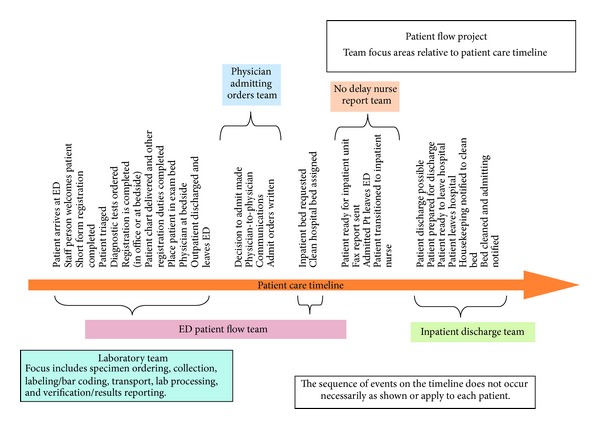
Patient flow diagram.

To accomplish this, we developed a patient care timeline for each ED visit (Figure 1) and created five interdisciplinary teams to focus on evaluating and improving different areas of care. The teams and their missions are outlined as follows.

1. *ED Patient Flow.* Minimizing the time that patients spend in the ED through the application of best practices.
2. *Laboratory Turnaround Time.* Managing the ordering, collecting, testing, and verification of lab work through improved and standardized procedures.
3. *No Delay Nurse Report.* Transfer of admitted patients to inpatient unit within 30 minutes of ED Nurse's report.
4. *Physician Admitting Orders.* Expediting completion of admitting orders by inpatient team for admitted patients.
5. *Inpatient Discharges.* Decreasing inpatient length of stay through effective discharge planning activities.

Each team studied the patient's experience at different points in the care timeline, identified the bottlenecks, and developed a plan of action. Teams met weekly and reported to the project leadership biweekly. We organized quarterly daylong summits for teams to present their findings, progress, and recommendations. Every team's plan of action recommendation was vetted by all five teams and implemented immediately after majority approval. Eighteen months after the start of the project, we held a final summit which marked the end of the analysis and recommendation phase.

The overall recommendations from the teams were to implement front-end reengineering, improve throughput and expedite patient disposition. Reengineering the arrival phase of the ED patient experience included the creation of the “Patient Partner” role, establishing a rapid assessment (RA) unit, and instituting bedside registration.

Due to the diversity of our patient population, we hired nonclinical multilingual persons with customer service background to fulfill this important role. Our Patient Partners welcomed and greeted patients, performed a miniregistration, generated a patient encounter in the electronic medical record, and escorted the patient immediately to a RA bed.

We simplified our initial registration process to the bare minimum in order to identify the patient's electronic health record (EHR) or create one for a new patient in the most expeditious way. This new miniregistration consisted of three questions: name, social security number (or date of birth), and chief complaint. Eventually, during downtime between tests and procedures, full bedside registration was performed after nursing and physician assessment, patient stabilization, and initiation of patient care.

After arrival and miniregistration, patients were immediately escorted into the RA unit where they met our clinicians. The RA unit combined the space, resources, and personnel of the previous registration, triage, and express care areas. The purpose of this unit was to facilitate rapid assessment and treatment at the point of entry to the ED. We augmented space and staffing, minimized redundancies, and created parallel functions without adding any new resources. Our plan was to triage patients in accordance with the national average of 7 minutes using emergency severity index (ESI) triage protocol. In the RA unit, low acuity patients (ESI 4 and 5) would get their complete care in the RA unit, without ever entering the acute ED area. Patients triaged with the more concerning ESI levels (1, 2, and 3) would be immediately moved to the acute ED areas where they could be evaluated and treated by the clinical team.

Improving ED throughput involved keeping all beds full while allowing specialized areas to be flexible. To this end, all beds became telemetry capable and we loosened up the rigid designations of specialized areas (adult, pediatric, trauma, psychiatric, etc.) so that any patient could be evaluated in any bed during high census intervals in order to maximize bed utilization. We also empowered the Charge Nurse to be the gatekeeper for patient flow in the department by problem solving during volume surges, expediting bed access for new patients, and coordinating timely assessment, treatment, and disposition of patients with the clinical teams.

As we examined the ED work processes for inefficiencies, we found a lack of clarity in staff roles including many gaps and overlap in their responsibilities. While this was probably a consequence of the need to multitask and collaborate in the ED, we found that this led to unclear accountability, which in turn resulted in redundancies, poor communication, medical errors, and delay of care. Consequently, we defined and clarified the roles of all ED personnel. We also trained them to perform tasks that were otherwise done by visiting specialized staff like phlebotomy and respiratory therapy. The laboratory team additionally focused on improving diagnostic turnaround times.

It is common knowledge that Emergency Department crowding is directly related to the total length of stay and boarding of admitted patients. To that end, we had a high focus on expediting the disposition of admitted patients and reducing their ED total length of stay. One of the interventions that we implemented was early identification of patients with high likelihood of being admitted even before all ancillary data was available. We created a special status for these patients on our electronic ED tracking board and made it accessible to the hospital admitting service, the hospitalists, the residents, and nurses on the inpatient floors, housekeeping, and transport. This provided an early warning process to all stakeholders that could affect the flow of admitted patients and allowed the admitting service time to prepare and receive the admission. 

We also developed methods to facilitate nursing and physician handoffs of admitted patients. For nursing, we implemented a faxed nursing report handoff. This report format was developed jointly by the ED and inpatient nurses and included all pertinent clinical and nonclinical information and was used on all noncritical patients. Once the patient was ready to be sent to the inpatient floor, several actions occur in order: the ED nurse would hand the completed nursing report to the ED secretary who would in turn fax it to the inpatient unit and call to verify that it had been received. The nurse on the inpatient unit receiving the patient would review the report and call the ED with any questions or concerns. The patient was then transported to the floor within 30 minutes of faxing the report unless valid concerns were communicated by the inpatient staff. The faxed report was ultimately eliminated in 2011 when the inpatient service implemented the electronic medical record (EPIC) and was able to visualize the ED record in real-time.

Physician hand-off was reduced to one call from the ED to a single lead hospitalist who was designated on a daily basis to receive all communications from the ED regarding medical admissions. At the end of the call, the ED provider and hospitalist agreed on the patient diagnosis and designated an admitting attending and resident who were responsible for patient care, the level of care necessary (e.g., ICU, telemetry, or medical), the admission status (e.g., “inpatient” or “observation”), and any other special needs for the patient (e.g., “isolation” or “watch”). The lead hospitalist would communicate the information to the chosen admission team. This team was encouraged to call the ED with any questions or concerns otherwise they would complete the admission workup once the patient was transported to the inpatient unit.

Other interventions included early recognition and discharge of patients from the inpatient units to create beds for the ED particularly during high census periods. These interventions were supported by an institution wide escalation process to notify all stakeholders and leaders of critical ED boarding situations and recruit their assistance to find solutions in real-time. Additionally, we instituted an institutional internal rapid response plan (Code Help) to provide the ED with additional resources to safely care for patients during high ED volume times and decompress the ED policy that allows inpatient units to receive ED patients. When activated, Code Help requires that all ED admitted patients would be transported to the inpatient units within 30 minutes even if they have to be temporarily treated in inpatient unit hallways.

### 2.1. Data Collection and Processing

The data was collected using the electronic medical record systems (Meditech and EPIC). Timestamps were used to compute the total length of stay time. Flags and patient records were used to determine whether a patient left without being seen (LWBS). Patient records were reviewed to access if acute myocardial infarction (AMI) and community acquired pneumonia (CAP) patients met the appropriate quality core measures. Patient Satisfaction Surveys were sent and data compiled by Press Ganey associates.

### 2.2. Primary Data Analysis

For our data analysis, we used a two-sample independent *t*-test to compare the mean of the “before” data, from January 2005 (FY05-Q1) to June 2006 (FY06-Q2) to the mean of the “after” data, from April 2008 (FY08-Q2) to December 2011 (FY11-Q4), of the following parameters: (1) median ambulance hours on diversion per fiscal quarter, (2) Press Ganey Patient Satisfaction Percentile scores, (3) median ED total length of stay time, (4) median door-to-provider time (or “ED wait time”), (5) quality core measurements (such as AMI and CAP), and (6) percent of volume that left without being seen (LWBS). The data generated between May 2006 (FY06-Q3) and March 2008 (FY08-Q1) (i.e., the “during data”) was not included in the analysis to decrease confounding; as during this time, the patient flow project was being implemented, and there were many changes occurring simultaneously. The “after” data collection began once all changes had been completely implemented. Equal variances were not assumed. We used the SPSS program for this analysis.

## 3. Results

**Figure 2:**
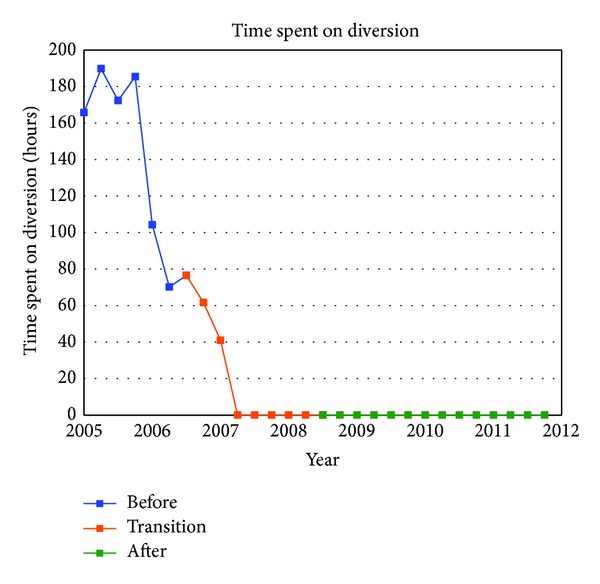
Hours of ambulance diversion. The quarterly number of hours the ED spent on diversion (refusing to receive ambulances).

**Figure 3:**
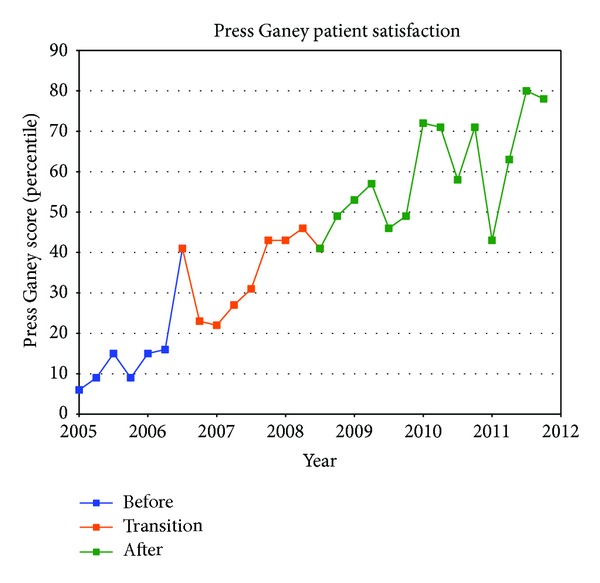
Quarterly patient satisfaction percentile scores based on Press Ganey report.

**Figure 4:**
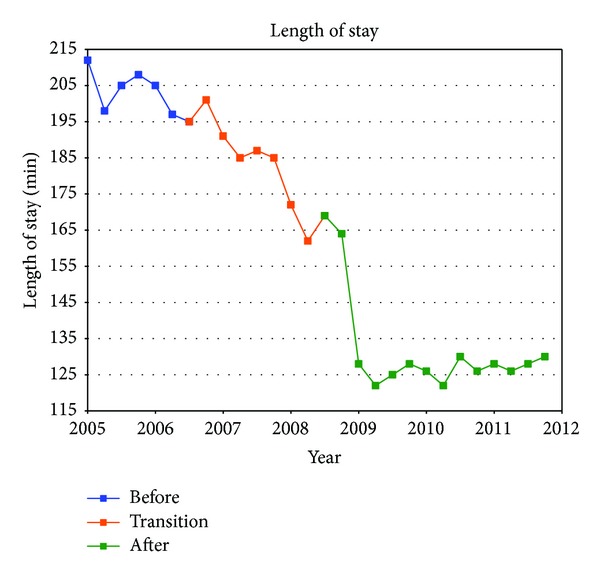
CHA ED total length of stay: the median time a patient spent from arrival to discharge.

**Figure 5:**
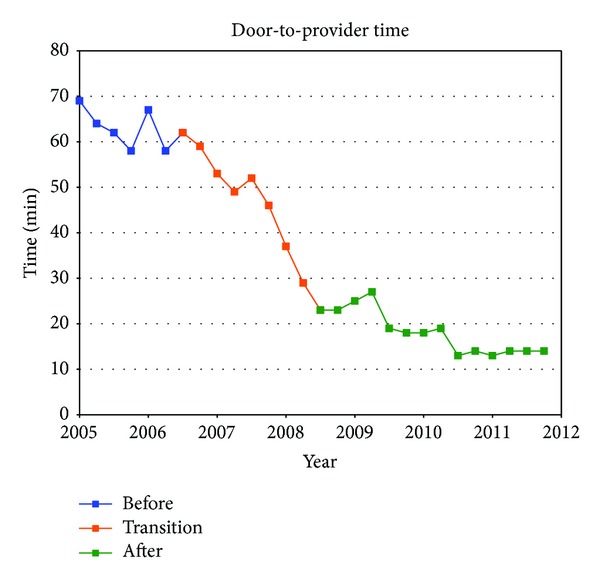
Median quarterly door-to-provider time: the median time patients spent waiting before being seen by a provider.

**Figure 6:**
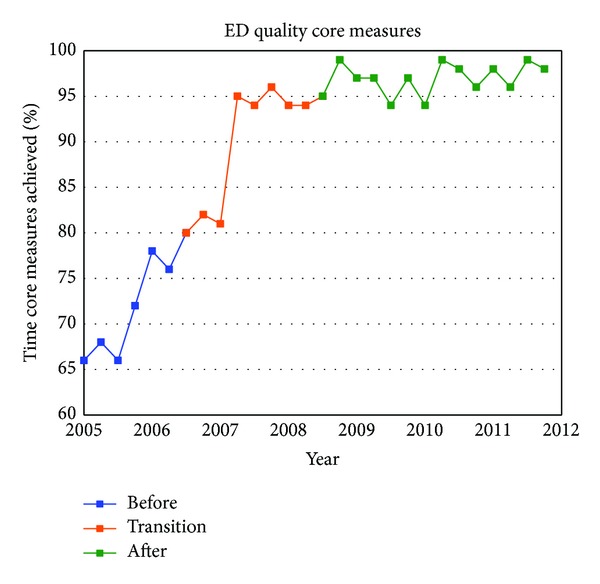
Core Measures: average ED sensitivity quality core measures indicator rates for two conditions. (1) Acute myocardial infarction (AMI): percent of time in which the patient received aspirin and beta-blocker on arrival. (2) Community acquired pneumonia (CAP): percent of time in which the patient received antibiotics within 4 hours and a blood culture prior to administration of antibiotics.

**Figure 7:**
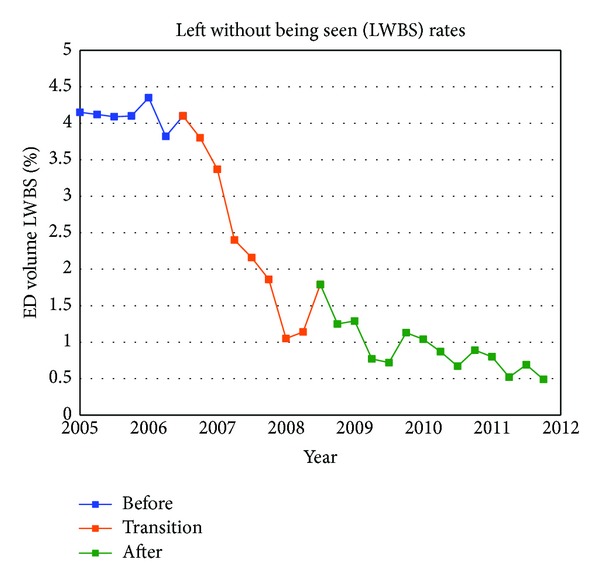
Left without being seen (LWBS) rate: percentage of patients who arrived at the ED but then left without receiving medical care.

**Figure 8:**
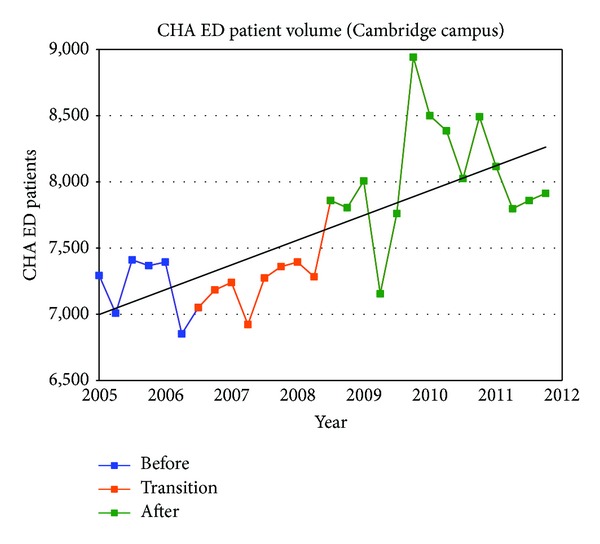
ED volume.

Table 1 summarizes the quarterly data for the study duration. The ED operational changes have had a significant positive impact on all measured metrics (Table 2). Ambulance diversion decreased from a record high mean of 148 hours per fiscal quarter before changes to 0 hours since April 2007 (Figure 2). Press Ganey Patient satisfaction scores rose from 12th percentile in 2005 to 59th percentile after implementation (Figure 3). ED total length of stay decreased from a mean of 204 minutes to mean of 132 minutes (Figure 4). Door-to-provider time decreased from a mean of 63 minutes to mean of 18 minutes (Figure 5). Compliance with ED specific quality core measures improved from a mean of 71% to 97% (Figure 6). The mean rate of ED patients that left without being seen (before treatment) treatment was completely dropped from 4.1% to 0.9% (Figure 7). All improvements were statistically significant with a *P* ≤ 0.001 (Table 2). More importantly these improvements occurred and were sustained amidst an 11% increase (from a mean of 7221 to 8044) in quarterly patient volume between 2005 and 2011 (Figure 8).

## 4. Discussion

TCH struggled with ED operations for many years and continued to fall short on many performance metrics. Prior to this initiative, diversions were routine, patient satisfaction scores were among the worst in the state, and there was a culture of inefficiency. In 2005, TCH spent more than 700 hours on diversion, scored in the 6th percentile in Patient Satisfaction, and had more than 4% of patients leave prior to receiving care (LWBS). Meanwhile, the ED was meeting quality core indicator rates for acute myocardial infarction (aspirin and a beta-blocker on arrival) and community acquired pneumonia (blood culture and appropriate antibiotics within 4 hours) less than 70% of the time.

Since the ED often served as first interaction between the hospital and its patients, poor ED performance affected institutional reputation and contributed to the stagnant ED volume levels. Since the ED accounts for more than 60% of all inpatient admissions, operational struggles were impacting the total patient experience for admitted patients. Improving the patient experience and quality of care in the ED was essential.

Recent publications regarding improving ED flow have shown that rapid assessment zone (RAZ), advanced triage protocols, and tracking systems/whiteboards resulted in a significant reduction in both total length of stay and left without being seen rates [8–14]. Furthermore, the utilization of the “Lean” principles of the Toyota Production system greatly enhanced efforts to improve throughput time and quality assurance [9–11]. A cohesive staffing strategy and concrete flow targets were also crucial components of the ED redesign [15–20].

Through a comprehensive assessment process, the ED management team decided to address these issues by optimizing front-end operations and utilizing a team-wide approach to improve patient throughput and disposition. In our front end reengineering, we effectively eliminated our sequential intake process where patients went through extended triage and a comprehensive registration before entering the ED and receiving care. Instead, we created a process where the patient was greeted by a customer service expert and placed in an ED bed immediately after a three-question miniregistration to identify the patient's electronic health record (EHR) or create one for a new patient in the most expeditious way. Once inside, patients were evaluated by any member of the care team that was available and their care was initiated. Full registration took place after nursing and physician assessment, stabilization, and initiation of care. Currently over 70% of ED patients in both RA and main ED stay in the same room and are cared for by the same physician/nursing team throughout their ED stay. We found that this approach served to limit the number of times a patient was moved, the number of times they had to present their story, the number of nursing and physician handoffs, the likelihood of miscommunication and errors, and the total length of stay. After implementation of these initiatives, we saw statistically significant improvement in all metrics including zero diversion since April 2007, two years before diversion was banned in Massachusetts. With collaboration and inclusion, we were able to make strides in staff alignment and performance achievements in a system that included 14 distinct unions representing various disciplines.

Emergency Department patient flow is difficult to transform for many reasons including culture and history. Traditionally, patient flow is based on inefficient processes leading staff to become myopic on single tasks, causing patients to repeat information to multiple clinicians, and producing undesirable outcomes like boarding, diversion, and long waits which all work to increase the number of patients leaving without being seen. Part of the reason why so many hospital EDs follow such hopelessly inefficient patient flow standards has been the ambivalence thinking that ED patterns are intractable and cannot be solved without substantial capital infusion to expand facilities and the addition of staff. We have discovered that ED transformation is manageable, strategic, and inexpensive. Along with the new operational changes came new structures for accountability and communication that allowed our team to manage in fundamentally different ways. We followed a plan that allowed for quick victories while continuing a gradual trend of overall change to improve our operations.

We believe the three main factors that led to our success were gaining administrative support, aligning a leadership team, and stakeholder inclusion. The Emergency Department is unique in that it interfaces with all other departments within the institution and even some services offered outside of the institution. Before we began to tackle the Emergency Department pitfalls, it was necessary to gain administrative support for such a project. This support was crucial to resolve ED throughput obstacles surrounding physician and nursing handoff of admitted patients and the ability to institute our Code Help policy to decompress the ED at times of high volume. The creation of Code Help and the knowledge that a full inpatient unit will be receiving admitted patients to its hallways motivated the staff in receiving units to work harder on their discharges.

During times of change, confusion among staff and unclear leadership is a recipe for failure. The key to any success is to have a well-developed mission that has the total support of the physician, nursing, and administrative ED leadership. We created a concrete mission statement and made certain that every person in the department, administrators, nurses, and physicians alike, across all three locations, knew what we wanted to accomplish and why changes were necessary. We also realized that a frontline staff participation and inclusion of all stakeholders was critical to the adoption and acceptance of any new initiative.

In summary, inefficiencies in the ED throughput process and delays of care may negatively impact patient satisfaction and patient outcomes [20–24]. During our operations overhaul we tackled this problem by improving the ED flow process, changing the staff culture, and placing the patient first. Ultimately, the Cambridge ED was able to meet and sustain our target outcomes and goals. We became a best practice institution based on patient satisfaction, reduced the door-to-provider time, and increased total ED volume and capacity. Improving ED operational efficiency allowed us to accommodate increasing volume while at the same time improving the quality of care and satisfaction of ED patients. This implementation serves to demonstrate that outcomes and cultural traditions can be improved through strategy rather than heavy capital investment.

## Figures and Tables

**Table 1:** Raw data used for statistical analysis, collected from January 2005 to December 2011.

Time	Median patient satisfaction per quarter (Press Ganey percentile)	Median door-to-provider time per quarter (in minutes)	Median total length of stay per quarter (in minutes)	Total hours on diversion per quarter	Percentage successfully meeting core measures per quarter	Left without being seen median rate per quarter (%)	ED Volume per quarter
Before							
FY05-Q1	6	69	212	165.79	66	4.15	7,293
FY05-Q2	9	64	198	189.78	68	4.12	7,008
FY05-Q3	15	62	205	172.33	66	4.09	7,411
FY05-Q4	9	58	208	185.42	72	4.1	7,367
FY06-Q1	15	67	205	104.31	78	4.35	7,394
FY06-Q2	16	58	197	70.21	76	3.82	6,852
During							
FY06-Q3	41	62	195	76.53	80	4.1	7,051
FY06-Q4	23	59	201	61.70	82	3.9	7,184
FY07-Q1	22	53	191	40.99	81	3.37	7,240
FY07-Q2	27	49	185	0.00	95	2.4	6,922
FY07-Q3	31	52	187	0.00	94	2.16	7,274
FY07-Q4	43	46	185	0.00	96	1.86	7,360
FY08-Q1	43	37	172	0.00	94	1.05	7,394
After							
FY08-Q2	46	29	162	0.00	94	1.14	7,283
FY08-Q3	41	23	169	0.00	95	1.79	7,859
FY08-Q4	49	23	164	0.00	99	1.25	7,805
FY09-Q1	53	25	128	0.00	97	1.29	8,007
FY09-Q2	57	27	122	0.00	97	0.77	7,155
FY09-Q3	46	19	125	0.00	94	0.72	7,761
FY09-Q4	49	18	128	0.00	97	1.13	8,942
FY10-Q1	72	18	126	0.00	94	1.04	8,500
FY10-Q2	71	19	122	0.00	99	0.87	8,386
FY10-Q3	58	13	130	0.00	98	0.67	8,025
FY10-Q4	71	14	126	0.00	96	0.89	8,492
FY11-Q1	43	13	128	0.00	98	0.8	8,116
FY11-Q2	63	14	126	0.00	96	0.52	7,797
FY11-Q3	80	14	128	0.00	99	0.69	7,859
FY11-Q4	78	14	130	0.00	98	0.49	7,913

FY: Fiscal year; Q: quarter.

**Table 2:** Summary of results and statistical analysis.

Metric	Mean		*P* value	Mean difference	95% Confidence interval of the mean difference
	Before* (*N* = 6)	After** (*N* = 14)			
Door-to-provider time	63	18.14	<0.001	44.86	(39.83, 49.89)
Total length of stay	204.17	132.29	<0.001	71.88	(62.24, 81.52)
ED hours on diversion	147.97	0	<0.001	147.97	(96.53, 199.41)
Core measures (%)	71	96.93	<0.001	25.93	(20.15, 31.35)
Left without being seen (%)	4.11	0.92	<0.001	3.18	(2.94, 3.43)
Quarterly volume	7220.83	8044.07	<0.001	823.24	(506.93, 1139.55)
Patient satisfaction Press Ganey (%ile)	11.67	59.36	<0.001	47.69	(38.44, 55.95)

*Before: FY05-Q1 to FY06-Q2.

**After: FY08-Q3 to FY11-Q4.

## Abbreviations and Acronyms

AMI: Acute myocardial infarction
TCH: The Cambridge Hospital
CAP: Community acquired pneumonia
ED: Emergency Department
FY: Fiscal year
LWBS: Left without being seen
RA: Rapid assessment
TLOS: Total length of stay.

## Limitations

These changes were implemented in a medium-sized, urban ED and some of the initiatives described and results derived may not be applicable to EDs operating under different constraints and with different patient populations. The institution of the electronic health record in the ED was another confounding factor. However, we feel that the changes implemented in the TCH ED would be beneficial to many EDs facing the challenges of crowding in times of limited resources.
